# Packaging materials with desired mechanical and barrier properties and full chemical recyclability

**DOI:** 10.1038/s41467-019-11525-x

**Published:** 2019-08-08

**Authors:** Ainara Sangroniz, Jian-Bo Zhu, Xiaoyan Tang, Agustin Etxeberria, Eugene Y.-X. Chen, Haritz Sardon

**Affiliations:** 1 POLYMAT, Department of Polymer Science and Technology, University of the Basque Country UPV/EHU, Manuel de Lardizabal, 3, 20018 Donostia, Spain; 2 Department of Chemistry, Colorado State University, Fort Collins, CO 80523-1872 USA

**Keywords:** Pollution remediation, Polymer characterization

## Abstract

Plastics have become indispensable in modern life and the material of choice in packaging applications, but they have also caused increasing plastic waste accumulation in oceans and landfills. Although there have been continuous efforts to develop biodegradable plastics, the mechanical and/or transport properties of these materials still need to be significantly improved to be suitable for replacing conventional plastic packaging materials. Here we report a class of biorenewable and degradable plastics, based on copolymers of γ-butyrolactone and its ring-fused derivative, with competitive permeability and elongation at break compared to commodity polymers and superior mechanical and transport properties to those of most promising biobased plastics. Importantly, these materials are designed with full chemical recyclability built into their performance with desired mechanical and barrier properties, thus representing a circular economy approach to plastic packaging materials.

## Abstract (web summary)

Biodegradable plastics usually have poorer mechanical or transport properties compared to conventional plastics. Here the authors show a class of biorenewable and chemically recyclable plastics based on copolymers of γ-butyrolactone and its ring-fused derivative, with competitive properties compared to conventional plastics.

## Introduction

Plastics are a material of choice in packaging applications because of their low cost, light weight, and high performance, coupled with good processability. It is expected that by 2050 the plastic packaging production will exceed 250 million metric tons^1^. Despite most of plastics employed in the packaging industry are used for less than a week, the durability, one of plastics’ greatest assets, is causing tremendous growth of disposed plastics as polluting waste. This growth, together with the fact that the vast majority of synthetic polymers are designed for performance and durability but not for degradability and recyclability, has brought millions of tons of plastic accumulation in the oceans and landfills^1^.

The main requirements for materials to be used in packaging are a good barrier character to water vapor and oxygen, good mechanical performance, and transparency; therefore, polymers have dominated the packaging market. Nowadays, the most widely used materials are poly(ethylene terephthalate) (PET) and polyolefins such as polyethylene (PE) and polypropylene (PP). Even with current practices of recycling, it is estimated that almost 95% of the plastic packaging materials value is not retained for subsequent uses after a short single use, representing an $80–120 billion annual loss to the economy^1^.

Several approaches have been investigated to address such plastics packaging waste problems, such as designing highly selective catalysts and unique chemistries^2,3^ to effectively depolymerize plastic materials into building blocks or monomers for polymer production, or designing additives for more effectively recycling mixed PE and isotactic PP materials into equal or possibly higher value materials^4^. In a short term, these approaches could be part of the solution to reduce the plastic waste, but for longer term implementation, questions such as economic viability of the process must be addressed and appropriate recycling strategies for the upcycled plastics must also be considered. Another strategy developed in the past two decades to address the end-of-life issues of packaging materials is the use of biodegradable polymers such as polylactide (PLA) or poly(3-hydroxybutyrate) (PHB), arguably the two most successful and extensively investigated examples. Biodegradable polymers are excellent alternatives to conventional petroleum-based, nondegradable plastics, as they are derived from biorenewable sources and can be enzymatically or hydrolytically degraded, thus leading to an environmentally closed circular ecosystem. However, the high permeability in the case of PLA and the poor mechanical properties in the case of PHB (a brittle material), plus their lack of high chemical recyclability^2^, have limited their potential^5^.

Beside these approaches, the design of plastics with recyclability built into their performance aiming for a fully plastic circular economy has been considered. With specifically designed monomers, reaction conditions can be used to select the direction of the monomer-polymer equilibrium or the closed-loop chemical cycle. Therefore, chemical recycling presents an attractive alternative since the polymer waste is employed to obtain the monomer for virgin polymer reproduction^6–18^, thus closing the loop and recovering the economic value of the post-consumer material. Recently several chemically recyclable polymers have been developed, such as polyesters^6–11^, polyurethanes^12^, and polycarbonates^13,14^. Among these materials those based on poly(γ-butyrolactone) (PγBL) core are highly interesting since they are obtained using renewable sources and can be fully recycled back to their monomers^7–11^. Moreover, being polyesters these materials have shown to hydrolytically degrade^19^ which, in case of escaping from sorting and collection of plastic that has reached the end of its life, will not accumulate in the environment.

In this work, we have investigated the potential of polyesters based on the PγBL core, which are specifically designed with full chemical recyclability built into their performance, as potentially fully recyclable plastics for packaging applications. First we have investigated homopolymers, PγBL, and poly(*trans*-hexahydrophthalide) (PT6HP)^9^ for an initial assessment of the potential of these homopolymers by measuring their permeability to different gases as well as their mechanical properties. After these initial investigations, we have specifically designed a copolymer, PT6HP-*co*-PγBL, which exhibits excellent barrier and mechanical properties comparable to commercial petroleum-based polymers used in packaging and superior in certain properties to the two most promising bio-based polymers to date, PLA and PHB.

## Results

### Mechanical and transport properties of homopolymers

At the outset, we first investigated the potential of PγBL and PT6HP for packaging applications. For the details about the synthesis of both homopolymers see Supplementary Methods and Supplementary Fig. 1 in Supplementary Information. The packaging materials must exhibit good barrier properties to atmospheric penetrants such as water vapor, oxygen, and carbon dioxide, as well as favorable mechanical performance such as good elongation at break (see Supplementary Note 1 and Supplementary Eqs. 1–3 for details about permeability measurements). To evaluate their potential, these two homopolymers were compared under the same conditions with four commercial polymers widely used in the packaging industry: PE (low density PE, LDPE), PET, PHB, and PLA (specifically semicrystalline PLLA), see Supplementary Table 1 for more details about the materials.

**Fig. 1:**
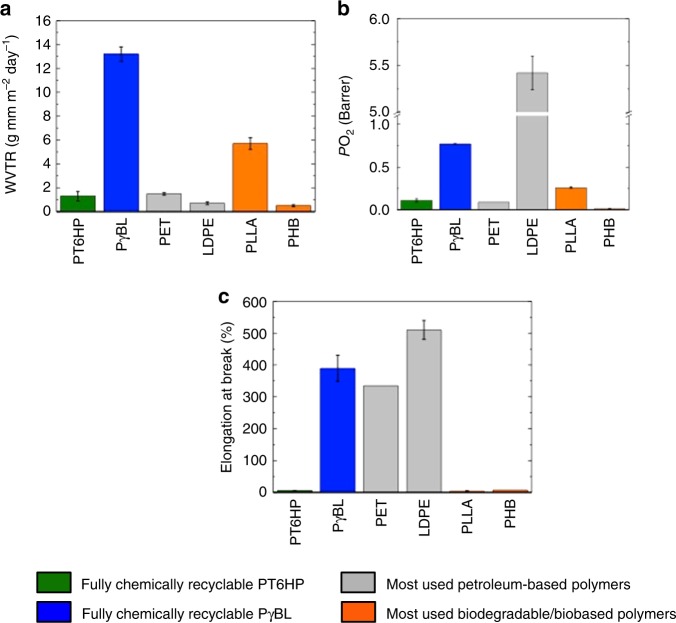
Transport and mechanical properties. **a** Water vapor transmission rate of fully chemically recyclable PT6HP and PγBL homopolymers and commercially available PET, LDPE and biobased/biodegradable PLLA and PHB. **b** Oxygen permeability coefficient of PT6HP, PγBL, PET, LDPE, PLLA, and PHB. **c** Elongation at break of PT6HP, PγBL, PET, LDPE, PLLA, and PHB. Error bars correspond to the standard deviation (s.d.) of at least four measurements

Mechanically, PγBL shows a ductile behavior with an elongation at break of >350%, (Supplementary Table 2), which is similar to PET^20^ and LDPE^21^. On the other hand, PT6HP shows a high tensile strength (39.4 ± 2.8 MPa, where 2.8 is the standard deviation) and Young’s modulus (3100 ± 300 MPa), but low elongation at break (5.1 ± 0.8%). It is worth noting that this elongation at break is similar to PLLA^22^ (3.6 ± 0.5%). In terms of transport properties, PγBL exhibited a high-water vapor transmission rate as well as high carbon dioxide and oxygen permeability values (Fig. 1 and Supplementary Table 3), thus undesirable for packaging. The poor barrier character of PγBL can be attributed to several factors, chiefly the low glass transition temperature (*T*_g_ = −45 °C).

On the other hand, PT6HP presents an outstanding barrier character since it shows a low-water vapor transmission rate (1.30 g mm m^−2^ day^−1^) and low oxygen (0.11 Barrer) and carbon dioxide (1.1 Barrer) permeability values. These results may arise from good chain packing of the rings in the main chain and higher *T*_g_ (49 °C). These permeability results are similar to those of PET^23^ (1.49 ± 0.11 g mm m^−2^ day^−1^ for water vapor, 0.09 ± 0.0 Barrer for oxygen, and 0.5 Barrer for carbon dioxide) and lower than LDPE (0.71 ± 0.12 g mm m^−2^ day^−1^ for water vapor, 5.42 ± 0.18 Barrer for oxygen, and 6.3 Barrer for carbon dioxide)^24^, widely employed in the packaging sector. The values are slightly higher than highly crystalline PHB (0.5 ± 0.08 g mm m^−2^ day^−1^ for water vapor and 0.01 ± 0.003 Barrer for oxygen)^22^, but significantly lower than PLLA (5.7 ± 0.5 g mm m^−2^ day for water vapor, 0.26 ± 0.01 Barrer for oxygen, and 1.2 Barrer for carbon dioxide)^22,25^. However, PHB, PLLA, and PT6HP present a major drawback: the lack of ductility (*vide supra*) that makes these materials undesirable for packaging applications.

The homopolymers have high transparency, especially PT6HP (see Supplementary Fig. 2), which is critical in many packaging applications. Overall, PT6HP showed excellent barrier properties to different penetrants but poor mechanical properties, while PγBL has ideal mechanical properties for packaging but poor barrier properties.

### Designing recyclable plastics for packaging applications

The above results obtained from the homopolymers provided a critical insight: combining the excellent barrier properties of PT6HP with the ductility of PγBL, while maintaining the recyclability should lead to a suitable chemically recyclable plastic for packaging applications. Thus, statistical aliphatic copolyesters of γBL and T6HP should be sought to optimize thermo-mechanical and gas barrier properties of related materials. However, the largely different reactivity of the two monomers (the thermodynamic polymerizability of T6HP, Δ*H*^°^_p_ = –20 kJ mol^−1^, is much higher than that of γBL, Δ*H*^°^_p_ = –5.1 kJ mol^−1^)^9^ makes the synthesis of random copolymers challenging.

Different types of catalysts have been employed to synthesize the copolymers. Common organic bases employed in ring-opening polymerization (ROP) reactions such as 1,8-diazabicycloundec-7-ene (DBU) and 1,5,7-triazabicicyclodec-5-ene (TBD) did not lead to copolymerization; instead, T6HP was isomerized to its *cis* isomer that is not polymerizable (Supplementary Table 4). Strong organic acids such as trifluoromethanesulfonic acid promoted polymerization, but the required long reaction time and high catalyst loading, plus the low monomer conversions achieved, make this catalyst not suitable for the copolymerization. To avoid the isomerization and also obtain higher conversions, coordination-insertion ROP catalysts such as La, Y, and Zn complexes were employed^26,27^ (Supplementary Fig. 1 and Supplementary Table 5). The yttrium catalyst supported by the tetradentate amino-bisphenolate ligand developed by Carpentier et al.^26^ showed the best results in terms of high molecular weights and good incorporations of both monomers in the copolymers.

Using this catalyst, copolymers with up to 27 mol% γBL were synthetized at room temperature, in bulk and a monomer/catalyst ratio of 1000/1. In all cases, high molecular weight copolymers were obtained with *M*_n_ values up to 347 kDa measured by gel-permeation chromatography (GPC) for the copolymer with 7% γBL incorporation. The incorporation of higher amounts of GBL led to lower molecular weights of 190 kDa and 52.0 kDa for copolymers with 18% γBL and 27% γBL incorporation, respectively. All the copolymers had a relatively low dispersity with *Đ* ~ 1.5 (Supplementary Table 6). The obtained molecular weights of the homopolymers are similar to those reported previously in literature^7–9,11^. In the case of copolymers the incorporation of γBL decreases the molecular weight, which is related to the nature of γBL since for the PγBL homopolymer low molecular weights are obtained.

The copolymer composition and the randomness of the copolymers were analyzed by NMR spectroscopy (see Supplementary Note 2 for further details). The compositions of the copolymers varying from 7 to 27% γBL incorporations can be readily calculated by focusing on the α-methylene proton signal at 2.38 ppm for γBL units in the copolymers (Supplementary Figs. 3–12).

The randomness of the copolymers was confirmed by ^13^C NMR analysis. Specifically, in the 60–70 ppm region four signals can be distinguished: 67.4 ppm for the α-carbon next to the carbonyl (CH_2_COO) corresponding to γBL-T6HP dyad (the underline denotes the analyzed nucleus in that unit) and 67.1 ppm for the carbon of T6HP-T6HP dyad. In the PγBL units the carbon of the methylene next to the carbonyl (CH_2_COO) appeared at 63.5 ppm, which corresponds to γBL-γBL dyad and at 63.3 ppm that corresponds to the carbon of γBL-T6HP dyad. From these signals and employing the corresponding equation (see Supplementary Eqs. 4–6) the randomness character (*η*) can be calculated with values close to 1 in all cases, thus confirming the random character of the copolymers (Supplementary Table 7).

The random character of the copolymer has been further confirmed by differential scanning calorimetry (DSC) analysis. As expected in all cases a single *T*_g_ was observed and the value of the *T*_g_ is between the *T*_g_’s of the two homopolymers (Supplementary Table 8). Thus, while PT6HP and PγBL exhibit a *T*_g_ of 49 °C and −45 °C, respectively, the copolymers with 18 and 27% of γBL incorporation possess a *T*_g_ of 34 °C and 24 °C, respectively, which makes these copolymers interesting candidates for packaging since it is envisaged that the ductility of the copolymers will be enhanced.

While the incorporation of only 7% γBL did not lead to any significant improvement in the mechanical properties, the incorporation of 18 and 27% of γBL led to a drastic improvement in ductility: the elongation at break was increased considerably to 149% and 436%, respectively, without a significant loss in Young’s modulus (Supplementary Table 2). Therefore, these two copolymers exhibit a much better ductility than PLLA and PHB, being similar to PET^20^ and LDPE^21^.

**Fig. 2:**
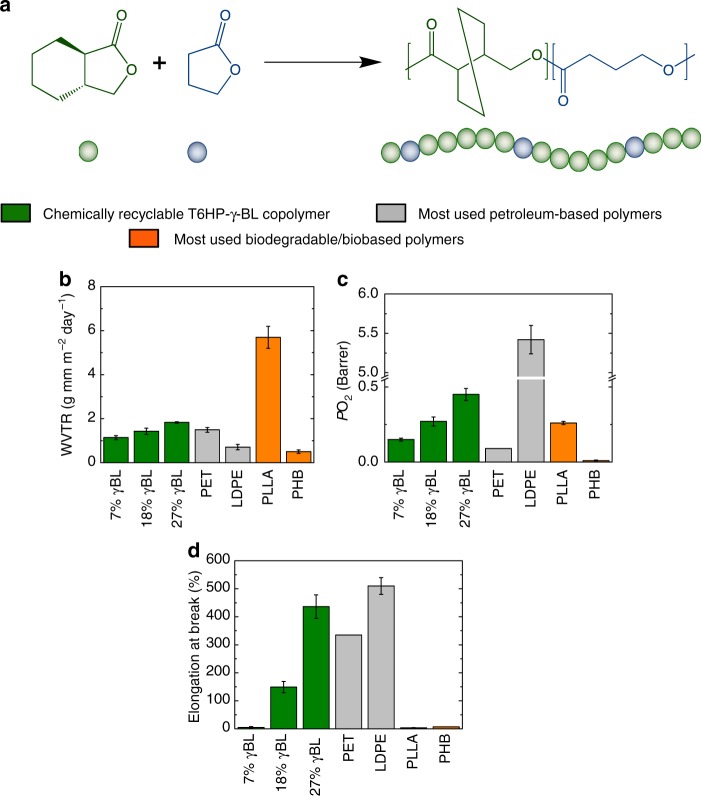
Synthesis of copolymers and their properties. **a** Schematic representation of copolymerization of T6HP and γBL. **b** Water vapor transmission rate of chemically recyclable copolymers and commercially available PET, LDPE and biobased/biodegradable PLLA and PHB. **c** Oxygen permeability coefficient of PT6HP, PγBL, PET, LDPE, PLLA, and PHB. **d** Elongation at break of PT6HP, PγBL, PET, LDPE, PLLA, and PHB. Error bars correspond to the standard deviation (s.d.) of at least four measurements

Next, the water vapor transmission rate, carbon dioxide, and oxygen permeabilities of these copolymers have been evaluated (Fig. 2 and Supplementary Table 3). The incorporation of 7% of γBL led to a WVTR value of 1.14 g mm m^−2^ day^1^, thus lower than PET (1.49 g mm m^−2^ day^−1^) and PLLA (5.7 g mm m^−2^ day^−1^). Copolymers with 18 and 27% γBL incorporations also gave great WVTR values of 1.43 and 1.83 g mm m^−2^ day^−1^, respectively. These values are similar to PET and three to five times smaller than PLLA, thus possessing a much better barrier character to water vapor as compared with PLLA.

The copolymers showed an excellent to moderate oxygen and carbon dioxide barrier character. In fact, the copolymer with 7% γBL incorporation exhibited an outstanding barrier character to oxygen with a low value of 0.15 Barrer. Copolymers with 18% γBL and 27% γBL incorporation gave oxygen permeability values of 0.27 and 0.45 Barrer, respectively, which are similar to PLLA and slightly higher than PET. It is worth noting that the oxygen permeability of these materials is 12–36 times smaller than LDPE, which makes these materials competitive with commodity polymers that are widely employed nowadays.

The permeability to carbon dioxide is moderate with permeability values of 1.0, 1.5, and 1.3 Barrer, respectively, for the three copolymers. These values are similar to PLLA (1.2 Barrer) and at least four times smaller than LDPE^28^ (6.3 Barrer). Therefore, these copolymers are good candidates to substitute PLLA and LDPE.

Overall, the copolymers showed ductility and barrier properties suitable for packaging applications, and they can be readily tuned by changing the copolymer composition to obtain materials suitable for each specific application. For example, the copolymer with 18% γBL incorporation showed good ductility and an outstanding barrier character to water vapor and moderate permeability to oxygen. Noteworthy is that this material possesses much better ductility and water barrier character relative to PLLA and LDPE, and the barrier character is similar to commercial PET.

### Full chemical recyclability of copolymers

**Fig. 3:**
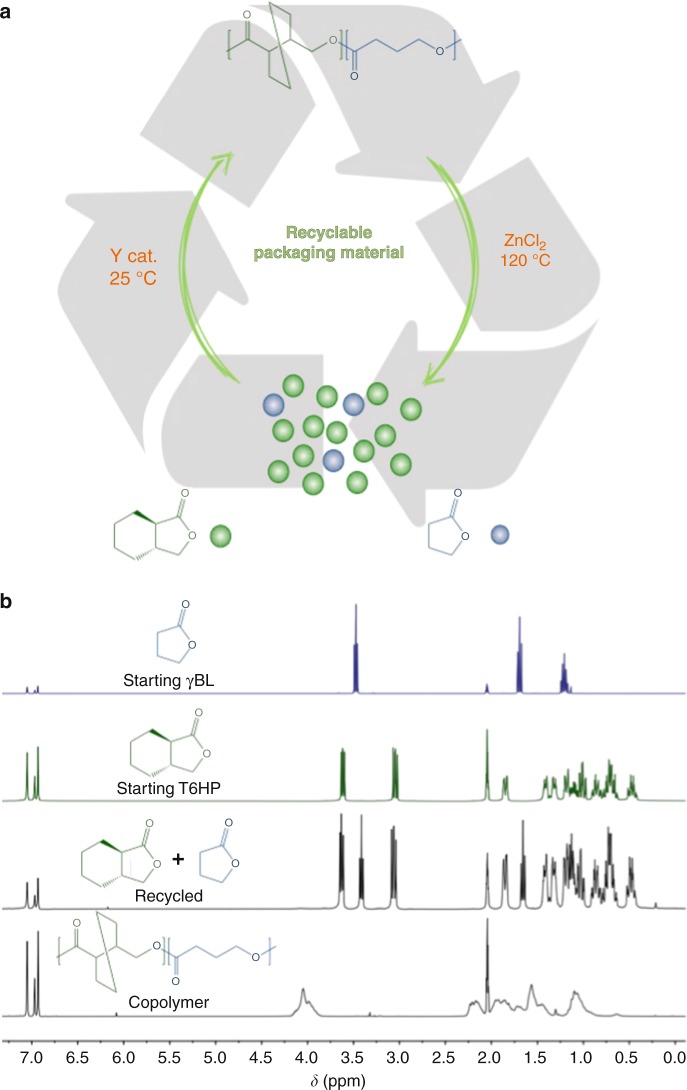
Chemical recyclability of copolymers. **a** Closed loop of chemically recyclable polymers. **b** ^1^H NMR spectra (C_7_D_8_) of starting γBL (top) and T6HP (second from top) comonomers as comparison, a mixture of the recycled comonomers after depolymerization (third from top), and the copolymer (27% γBL incorporation) before chemolysis (bottom)

As both the homopolymers of constituent comonomers, T6HP and γBL, exhibit full chemical recyclability^7,9^, we hypothesized that the copolymer consisting of these two monomers should also be completely recyclable by either thermolysis or catalyzed processes (chemolysis). Accordingly, the chemical recyclability of copolymer PT6HP-*co*-PγBL was investigated by chemolysis. The copolymer was heated at 120 °C for 60 h in the presence of a catalytic amount of ZnCl_2_ (2 mol%), cleanly recovering the two original monomers, T6HP and γBL in pure state (Fig. 3), therefore demonstrating the full chemical recyclability of the copolymer. The same depolymerization experiment was also done to a copolymer film, also achieving a full recovery of the comonomers (see the Supplementary Fig. 13).

## Discussion

Copolymers with excellent barrier and mechanical properties have been designed with chemical recyclability built into their performance as fully recyclable packaging materials. The results showed that with judiciously designed copolymer structures, it is possible to create chemically recyclable polymers that exhibit quantitative chemical recyclability and barrier properties comparable to petroleum-based PET and superior to biobased PLLA. These copolymers represent a promising class of materials that could be implemented in packaging applications with a closed-loop lifecycle through either their degradability or chemical recyclability, contributing toward the overarching goal of eliminating or diminishing plastic pollution.

## Methods

### Synthesis of copolymers

The general procedure for T6HP homopolymerizations and copolymerizations is described below. The reaction was carried out in 25 mL reactors at 25 °C in the glovebox under inert atmosphere. The catalyst was added to vigorously stirring monomers. After the adequate period of time, the reaction was stopped by immersing the reactor in liquid nitrogen and adding chloroform acidified with HCl (5%). The mixture was dissolved in chloroform and precipitated in excess cold methanol. The polymer was dissolved again in chloroform and precipitated in methanol three times to purify thoroughly and remove any catalyst and monomer residues. The isolated samples by filtration were dried in an oven under vacuum at 60 °C for 3 days until a constant weight was achieved.

### Synthesis of homopolymers

The synthesis of PT6HP homopolymer was performed in the same way as the copolymers, see above. The synthesis of PγBL was performed in 50-mL flame-dried Schlenk tubes interfaced to the dual-manifold Schlenk line using an external cooling bath. The reactor was charged with a predetermined amount of the yttrium catalyst and THF in the glovebox. The reactor was sealed, taken out of glovebox, and then immersed in a −40 °C cooling bath. After equilibration at the desired polymerization temperature for 10 min, the polymerization was initiated by rapid addition of monomer (17.2 g) via a gastight syringe ([γBL] = 10 mol L^−1^). After 12 h, the polymerization was quenched by addition of 30 mL of benzoic acid/CHCl_3_ (10 mg mL^−1^). The quenched mixture was then precipitated into 1000 mL of cold methanol, filtered, washed with methanol to remove unreacted monomer, and dried in a vacuum oven at room temperature to a constant weight; *M*_n_ = 42.2 kg mol^−1^, *Ð* = 1.90.

### Depolymerization procedure

A sealed J. Young type NMR tube was charged with PT6HP-*co*-PγBL copolymer (23 mg), ZnCl_2_ (2%), and deuterated toluene (0.6 mL) in a glovebox, under inert atmosphere. The NMR tube was sealed with a Teflon valve and taken out of the glovebox. The tube was immersed in a bath at 120 °C for 60 h and analyzed by NMR. Depolymerization of a copolymer film was also performed, following a similar procedure but using a Schlenk tube (see Supplementary Fig. 13).

### Spectroscopic characterization

NMR spectra were recorded on a Varian Inova 400 MHz (FT 400 MHz, ^1^H; 100 MHz, ^13^C) or a 500 MHz spectrometer.

### Molecular weight measurements

The molecular weight was measured employing GPC and depending on the samples and their solubility three different instruments were used.

A GPC instrument coupled with multi-angle light scattering employing an Agilent HPLC was fitted with one guard column and three gel permeation columns (PLgel 5 μm MIXED-C). The differential refractometer (TrEX) and light scattering detector (miniDAWN TREOS) employed are from Wyatt Technology. THF was used as eluent at a flow rate of 1.0 mL min^−1^. This equipment was employed for PT6HP-*co*-PγBL copolymers.

A Waters University 1500 GPC with one guard column and two columns (PLgel 5 μm mixed-C) was used for the measurements carried out at 40 °C and a flow rate of 1.0 mL min^−1^ using DMF as eluent. This equipment was employed to measure PγBL homopolymer.

A GPC instrument consisting of an Agilent HPLC system with one guard column and two columns (PLgel 5 μm mixed-C) was coupled with a Wyatt DAWN HELEOS II multi (18)-angle light scattering detector and a Wyatt Optilab Trex dRI detector. The measurements were performed at 40 °C employing chloroform as eluent. PT6HP was measured employing this equipment.

### Differential scanning calorimetry (DSC)

The characterization of the thermal properties was carried out by a differential scanning calorimeter from TA Instrument, model Q2000 V24. Approximately 3 mg sample was encapsulated in aluminum hermetic pans. A heating scan was performed from −80 °C to 200 °C at 10 °C min^−1^ heating rate, and the cooling rate was 10 °C min^−1^.

### Tensile tests

The mechanical properties were measured employing an Instron 5565 testing machine. The measurements were performed at 22 °C and a crosshead displacement rate of 5 mm min^−1^, except for the copolymer containing 27% γBL the crosshead displacement rate was 50 mm min^−1^. The specimens were cut according to ASTM D638 type 5 and they had a thickness of 40–80 μm. At least five specimens were tested for each reported value.

### Permeability measurements

Water vapor transmission rate was measured in a permeation cell at 25 °C according to ASTM E96-95 method. The cell, made of polytetrafluoroethylene, was partially filled with water and a polymeric membrane was placed above sealing its top. The measurements were performed in a Sartorius BP 210 D balance with 10^−5^ g readability and the mass loss was recorded in a computer. The reported values are at least the average of five measurements.

Oxygen permeability was measured by a Mocon OX-TRAN 2/21 MH equipment at 1 atm, 23 °C, and 0% relative humidity.

Carbon dioxide sorption was measured employing a Hiden IGA-2 electrobalance. The measurements were performed in the range of 1–20 bar and at 25 °C. After the adequate data treatment of the sorption kinetics, solubility, *S*, and diffusion coefficients, *D*, can be determined and, therefore, permeability, *P*, can be estimated.

## Supplementary information

Supplementary Information

## Data Availability

The authors declare that the data supporting the findings of this study are provided in the main article and the Supplementary Information.
