# Supplementary material for: Packaging materials with desired mechanical and barrier properties and full chemical recyclability
Source: Nat Commun. 2019 Aug 8;10:3559. doi: 10.1038/s41467-019-11525-x (PMC6687705; doi:10.1038/s41467-019-11525-x)
Supplement: Supplementary file 1 — Supplementary Information [file 41467_2019_11525_MOESM1_ESM.pdf]

Supplementary Information

Packaging materials with desired mechanical and barrier properties and full chemical recyclability

A. Sangroniz et al.

Supplementary Methods

Air and moisture sensitive materials were synthetized and manipulated in an inert gas filled glove box or in flamed Schlenk-type glassware on a dual-manifold Schlenk line, on a high vacuum line. The employed solvents and monomers were dried adequately. PT6HP monomer was synthetized according to literature[1]. Supplementary Figure 1 shows the organometallic catalysts employed in this work. Zinc and yttrium catalysts were synthetized according to literature[2,3].

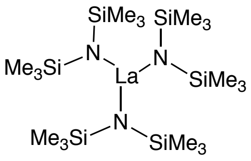

La

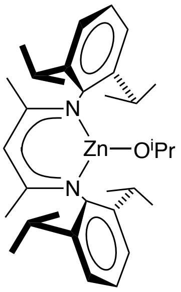

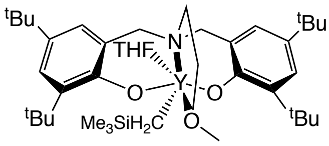

Y

Zn

Supplementary Figure 1. Chemical structure of different metallic catalysts employed. Tri[N,Nbis(trimethylsilyl)amide] lanthanum(Ш) (La) on the left, 2,6-diisopropylphenylsubstituted β-diiminate zinc isopropoxide complex (Zn) on the middle and tetradentate amino-bisphenolate yttrium alkyl complex (Y) on the right.

Supplementary Note 1 (Permeability measurements and data treatment)

Water vapor transmission rate (WVTR) was measured in a permeation cell at 25 °C according to ASTM E96-95 method. The cell, made of polytetrafluoroethylene, was partially filled with water and a polymeric membrane was placed above sealing its top. The measurements were performed in a Sartorius BP 210 D balance with 10^{-5} g readability and the mass loss was recorded in a computer[4]. The reported values were at least the average of 5 measurements.

The WVTR was calculated from the slope of the weight loss versus time employing Supplementary Equation 1,

$$WVTR = \frac{s \times l}{a \times \left(p_{ins} - p_{out}\right)} \tag{1}$$

where s is the slop of the plot, l is the thickness of the membrane, a is the area exposed to the water vapor and p_{ins} and p_{out} are the water vapor pressure inside and outside of the cell, respectively.

Oxygen permeability was measured by a Mocon OX-TRAN 2/21 MH equipment at 1 atm, 23 °C and 0 % relative humidity. The equipment provides directly the oxygen transmission rate that is related to permeability by the Supplementary Equation 2,

$$OTR\left(\frac{\text{cc mil}}{\text{m}^2\text{day}}\right) \times \frac{0.29398}{p\left(\text{mmHg}\right)} = P\left(\text{Barrer}\right) \tag{2}$$

Carbon dioxide sorption was measured employing a Hiden IGA-2 electrobalance. The measurements were performed in the range of 1-20 bar and at 25 °C. After the adequate data treatment of the sorption kinetics, solubility, S, and diffusion coefficients, D, can be determined[5]. Both, diffusion and solubility

coefficient are obtained from the data provided from the software and permeability can be estimated using the Supplementary Equation 3,

$$P = D \times S \qquad (3)$$

The supplier and characteristics of the commercial polymers employed for mechanical properties characterization and transport properties determination are reported in Supplementary Table 1.

Supplementary Table 1. Characteristics of commercial polymers employed

Material	M_w (g mol^{-1})	Supplier
PCL	49000	Polysciences, Inc.
PLLA	211100	Biomer
PHB	437000	Aldrich
PET	*	Brilen

* Intrinsic viscosity of 0.80 dL g^{-1} in *m*-cresol.

Supplementary Table 2. Mechanical properties of recyclable polymers vs commercial polymers for packaging

Polymers	Tensile strength at break (MPa)	Young modulus (MPa)	Elongation at break (%)
PT6HP	39.4 ± 2.8	3120 ± 300	5.1 ± 0.8
PT6HP-*co*-PγBL (7 %)	28.8 ± 2.4	3850 ± 500	5.0 ± 2.6
PT6HP-*co*-PγBL (18 %)	13.3 ± 1.2	2410 ± 210	149 ± 20
PT6HP-*co*-PγBL (27 %)	7.4 ± 0.6	640 ± 160	436 ± 42
PγBL	51.8 ± 5.0	660 ± 40	389 ± 41
PLLA[7]	54.2 ± 4.0	3520 ± 270	3.6 ± 0.5
PHB[7]	37 ± 5.0	5460 ± 340	0.7 ± 0.0
PET[8]	45	610	335
LDPE[9]	12 ± 0.4	88.4 ± 5.7	510 ± 30

Supplementary Table 3. Water vapor transmission rate, oxygen and carbon dioxide permeability values for homopolymers and copolymers

Samples	*WVTR* (g mm m^{-2} day^{-1})	*P* O_2 (Barrer)	*P* CO_2 (Barrer)
PT6HP	1.30 ± 0.40	0.11 ± 0.02	1.1
PT6HP-γBL 7 %	1.14 ± 0.09	0.15 ± 0.01	1.0
PT6HP-γBL 18 %	1.43 ± 0.14	0.27 ± 0.03	1.5
PT6HP-γBL 27 %	1.83 ± 0.03	0.45 ± 0.04	1.3
PγBL	13.2 ± 0.6	0.77 ± 0.001	6.8
PLLA[7,10]	5.7 ± 0.50	0.26 ± 0.01	1.2
PHB[7]	0.5 ± 0.08	0.01 ± 0.003	-
PET[11]	1.49 ± 0.11	0.09 ± 0.0	0.5
LDPE[12]	0.71 ± 0.12	5.42 ± 0.18	6.3

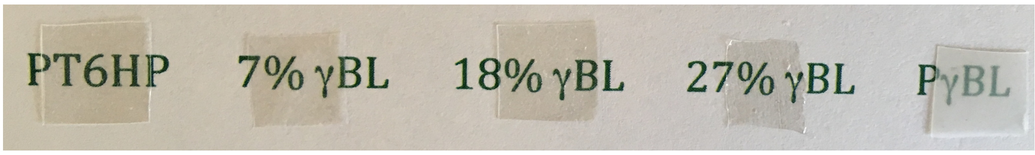

Supplementary Figure 2. Pictures of the homopolymer and copolymer films. PT6HP homopolymer in the left followed by PT6HP-γBL 7 %, PT6HP-γBL 18 %, PT6HP-γBL 27 % and PγBL.

Supplementary Table 4. Results of ring-opening copolymerization of T6HP and γBL employing organic catalysts

Run	Cat	M/Cat/I	Time (h)	Conv T6HP (%)	Conv γBL (%)
1	DBU	20/1/3	24	0	0
			72	0	0
			7 (days)	0	0
2	TBD	20/1/3	24	0	0
			72	0	0
			7 (days)	0	0
3	FMSA	10/1/2	24	21.6	5.3
			72	36.6	11.9
			7 (days)	55.8	18.1

General conditions: 400 mg, initiator = Ph_2CHCH_2OH, room temperature (≈ 25 °C). T6HP/γBL ratio 50/50. Monomer conversion was determined by ^1H NMR in $CDCl_3$.

Supplementary Table 5. Results of ring-opening copolymerization of T6HP and γBL employing different organometallic catalysts

Run	Catalyst	M/Cat	Conv T6HP (%)	Conv γBL (%)
1	La	1000/1	30.8	12.0
2	Zn	500/1	38.0	16.9
3	Y	1000/1	62.5	28.2

General conditions: 400 mg, room temperature (≈ 25 °C), T6HP/γBL ratio 50/50. Usually polymerization occurred in 30-60 min and ceased stirring, then the reaction was left without stirring for 6-8 hours. Monomer conversion was determined by ^{1}H NMR in $CDCl_3$.

Supplementary Table 6. Molecular weight and dispersity index of homopolymers and copolymers

Samples	M_w (kDa)	M_n (kDa)	$Đ$ (M_w/M_n)
PT6HP	1110	1018	1.09
PT6HP-*co*-PγBL 7 %	530	347	1.53
PT6HP-*co*-PγBL 18 %	286	190	1.51
PT6HP-*co*-PγBL 27 %	79.9	52.0	1.53
PγBL	80.2	42.2	1.90

Supplementary Note 2 (Composition and microstructure characterization of copolymers)

The compositions of the copolymers have been calculated by focusing on the α-methylene proton signal at 2.38 ppm for γBL units in the copolymers. Supplementary Equations 4-6 were employed to calculate the number-average sequence lengths (l_{i}) of T6HP and γBL building blocks and the randomness character (η)[6].

$$l_{T6HP} = \frac{2\left(\mathrm{T6HP}\right)}{\left(\mathrm{T6HP\text{-}}\gamma\mathrm{BL}\right)} \tag{4}$$

$$l_{\gamma BL} = \frac{2\left(\gamma\mathrm{BL}\right)}{\left(\mathrm{T6HP\text{-}}\gamma\mathrm{BL}\right)} \tag{5}$$

$$\eta = \frac{\left(\mathrm{T6HP} - \gamma\mathrm{BL}\right)}{2\left(\mathrm{T6HP}\right)\left(\gamma\mathrm{BL}\right)} \tag{6}$$

where (T6HP) and (γBL) are the two comonomer molar fractions and (T6HP-γBL) is the T6HP-γBL average dyad relative molar fraction.

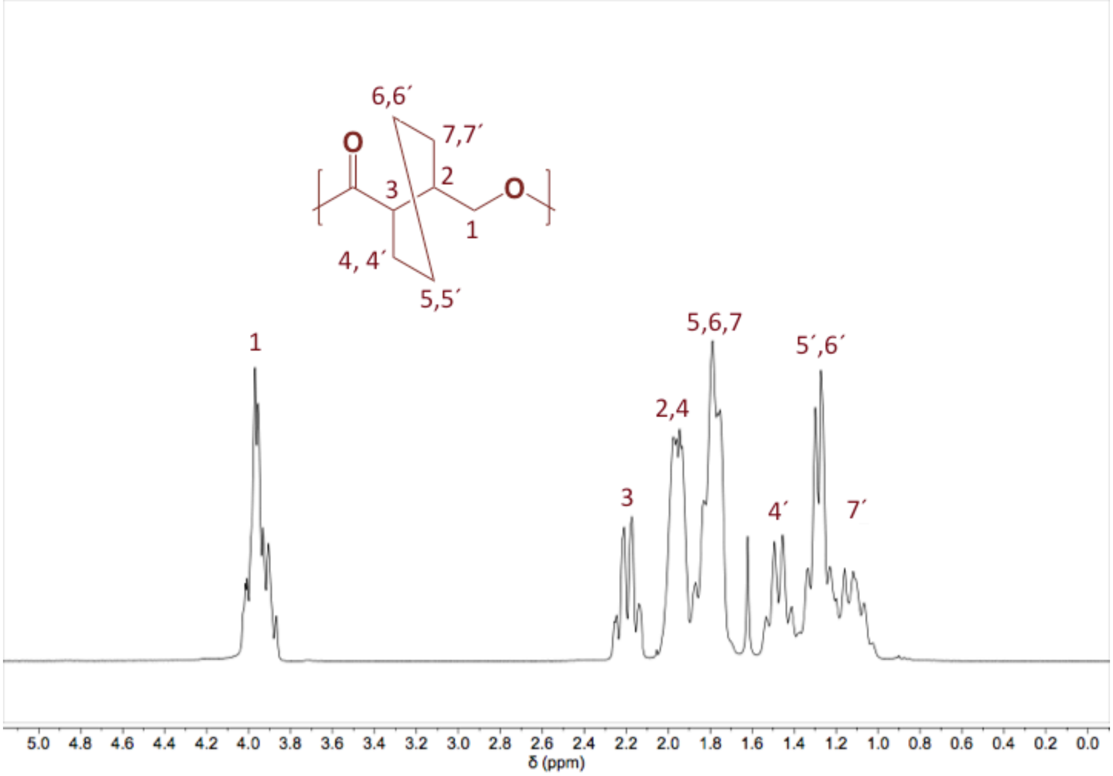

Supplementary Figure 3. ^{1}H NMR spectrum of PT6HP. Experimental conditions were as follows: 10 mg of sample, 25 °C, 3 s acquisition time, 1 s delay time, and 32 scans. The samples were dissolved in deuterated chloroform. The numbers on the signals correspond to the numbers in the chemical structure of the polymer.

As it can be seen in the spectra of PT6HP (Supplementary Fig. 3) the protons on the ring (labeled as 4, 5, 6 and 7) are diasterotopic; thus, the two protons attached to a carbon appeared at different resonances and were denoted with an apostrophe. Due to the complexity of the spectra the elucidation of the assignments has been carried out by performing different 2D spectra, see Supplementary Fig. 4 and 5.

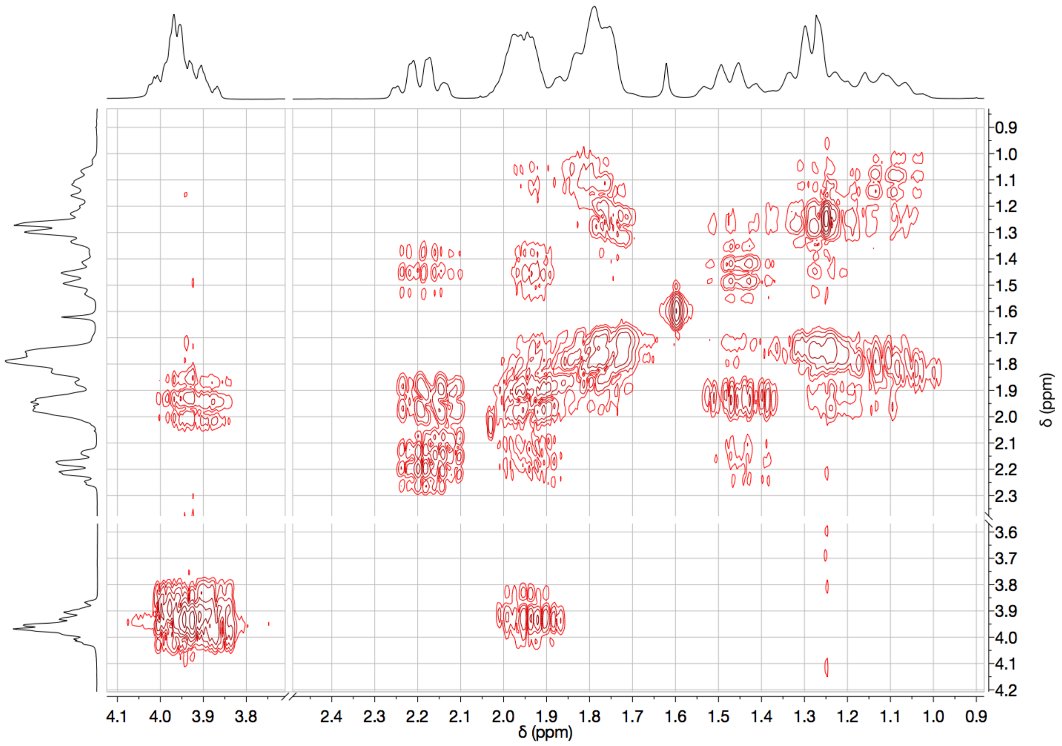

Supplementary Figure 4. 2D COSY NMR spectrum of PT6HP polymer. Experimental conditions were as follows: 10 mg of sample, 25 °C, 0.7 s acquisition time and 1.6 s delay time.

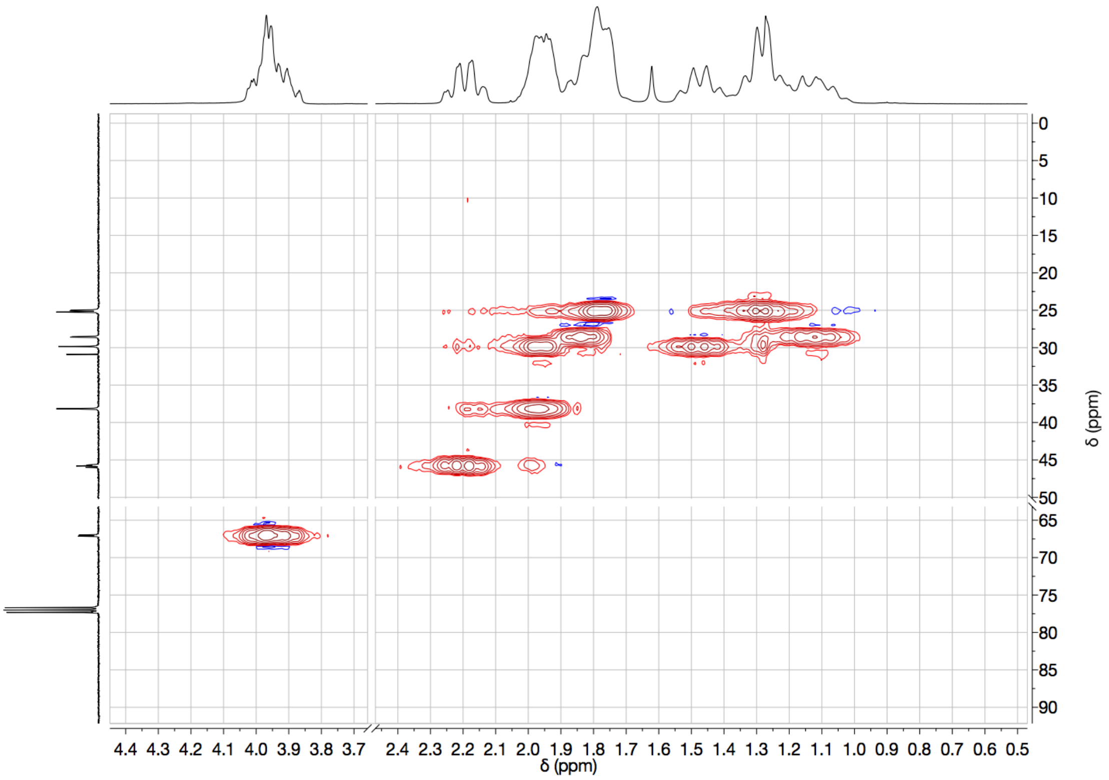

Supplementary Figure 5. 2D HSQC NMR spectrum of PT6HP polymer. The experiments were performed in deuterated chloroform and 25 °C. The spectral widths for the HSQC experiment were 2500 and 16611 Hz for the ^{1}H and the ^{13}C dimensions, respectively. The number of collected complex points was 2048 for the ^{1}H dimension. The number of transients was 64 and 256 time increments were recorded in the ^{13}C dimension. The $^{1}J_{CH}$ used was set as 145 Hz.

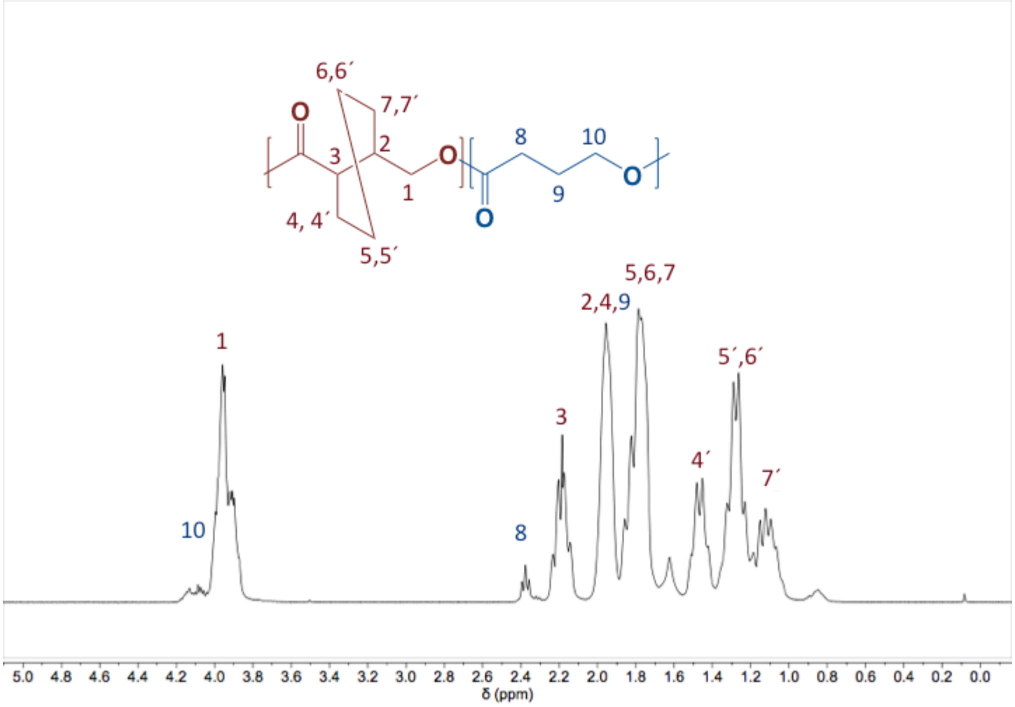

Supplementary Figure 6. ^{1}H NMR spectrum of PT6HP-*co*-PγBL 7 % γBL copolymer. Experimental conditions were as follows: 10 mg of sample, 25 °C, 3 s acquisition time, 1 s delay time, and 32 scans. The samples were dissolved in deuterated chloroform. The numbers on the signals correspond to the numbers in the chemical structure of the polymer.

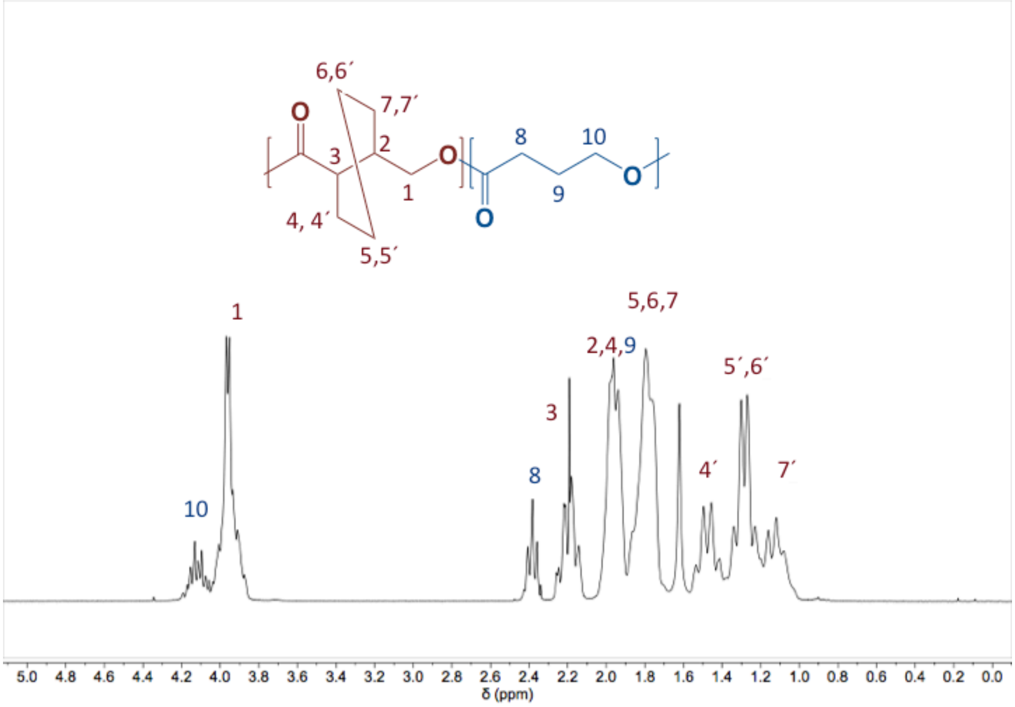

Supplementary Figure 7. ^{1}H NMR spectrum of PT6HP-*co*-PγBL 18 % γBL copolymer. Experimental conditions were as follows: 10 mg of sample, 25 °C, 3 s acquisition time, 1 s delay time, and 32 scans. The samples were dissolved in deuterated chloroform. The numbers on the signals correspond to the numbers in the chemical structure of the polymer.

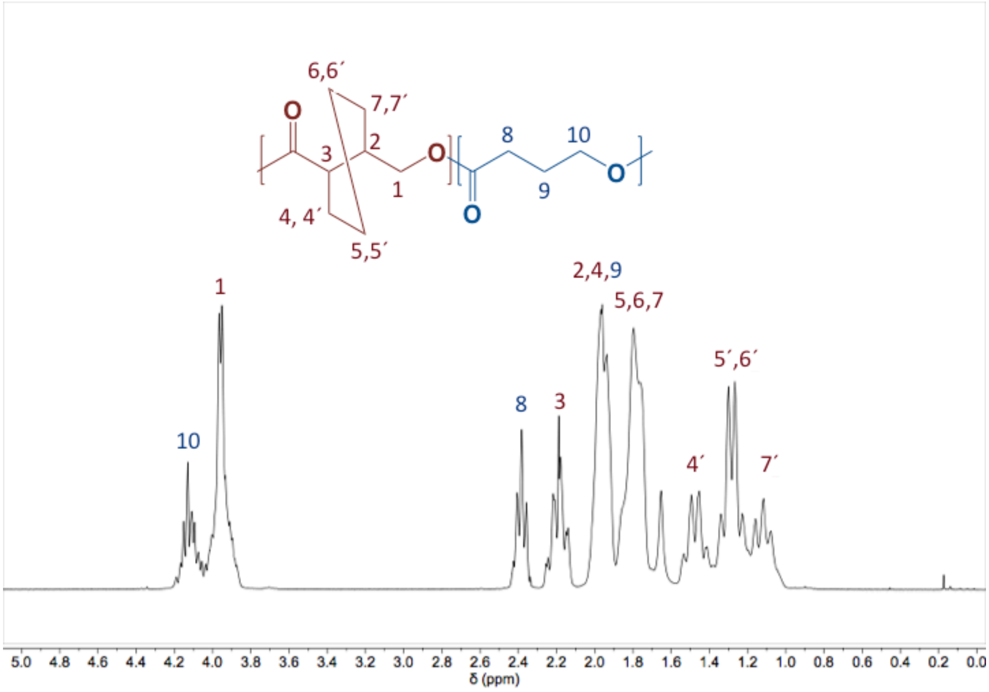

Supplementary Figure 8. ^{1}H NMR spectrum of PT6HP-*co*-PγBL 27 % γBL copolymer. Experimental conditions were as follows: 10 mg of sample, 25 °C, 3 s acquisition time, 1 s delay time, and 32 scans. The samples were dissolved in deuterated chloroform. The numbers on the signals correspond to the numbers in the chemical structure of the polymer.

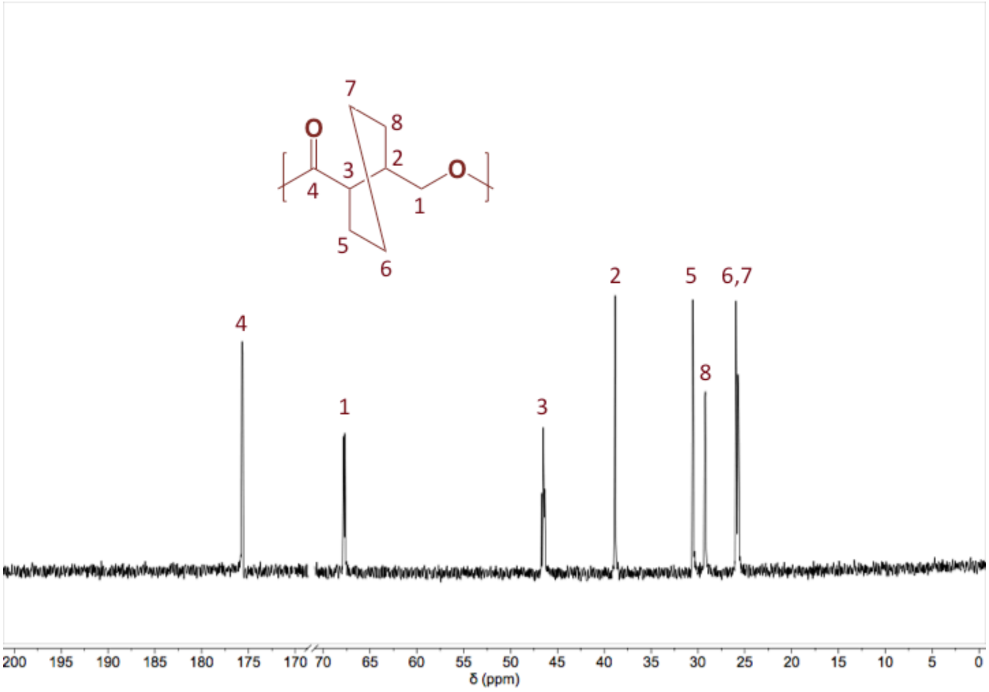

Supplementary Figure 9. ^{13}C NMR spectrum of PT6HP homopolymer. Experimental conditions were as follows: 40 mg of sample, 25 °C, 1 s acquisition time, 2 s delay time, 10 µs pulse, spectral width 25900 Hz and more than 1100 scans. The samples were dissolved in deuterated chloroform. The numbers on the signals correspond to the numbers given in the chemical structure of the polymer.

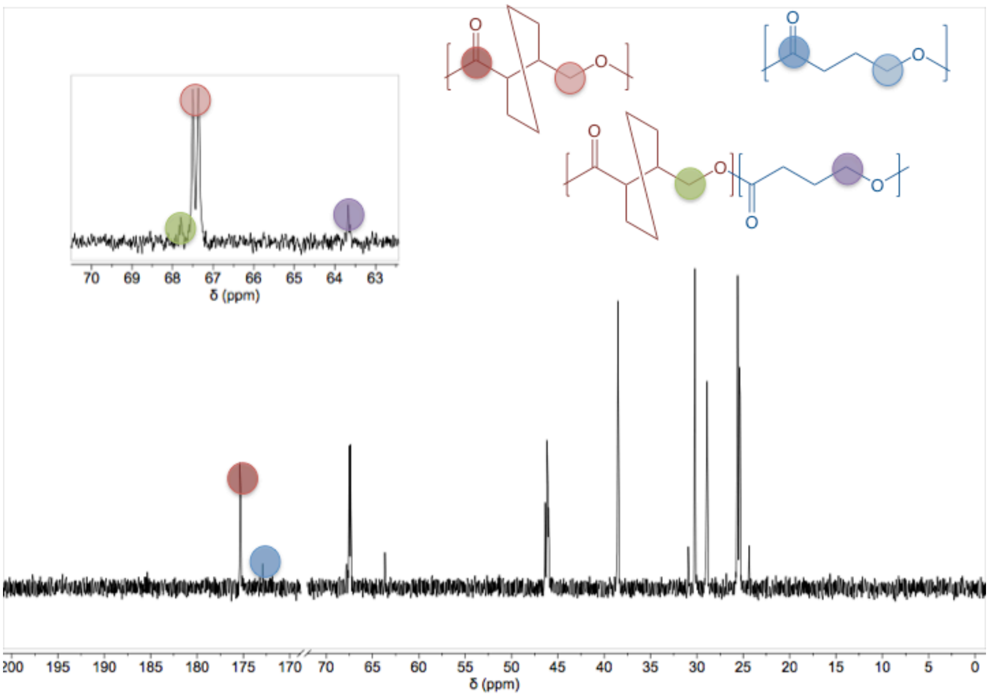

Supplementary Figure 10. ^{13}C NMR spectrum of PT6HP-*co*-PγBL 7 % γBL copolymer. Experimental conditions were as follows: 40 mg of sample, 25 °C, 1 s acquisition time, 2 s delay time, 10 μs pulse, spectral width 25900 Hz and more than 1100 scans. The samples were dissolved in deuterated chloroform. The colors in the signals correspond to the colors in the chemical structure and these signals are employed to elucidate the diads.

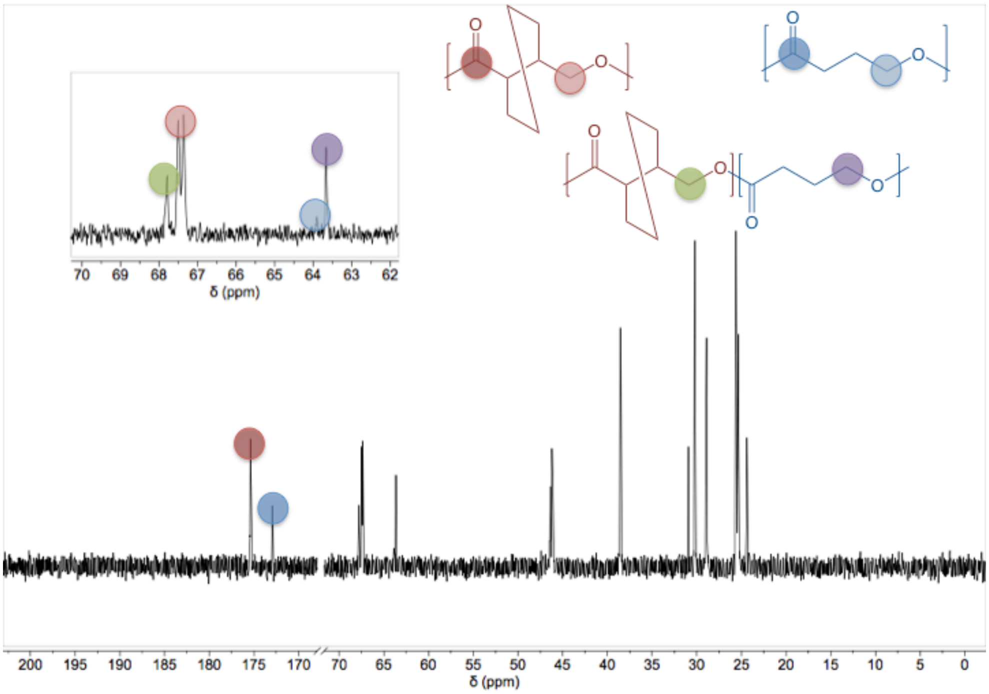

Supplementary Figure 11. ^{13}C NMR spectrum of PT6HP-*co*-PγBL 18 % γBL copolymer. Experimental conditions were as follows: 40 mg of sample, 25 °C, 1 s acquisition time, 2 s delay time, 10 μs pulse, spectral width 25900 Hz and more than 1100 scans. The samples were dissolved in deuterated chloroform. The colors in the signals correspond to the colors in the chemical structure and these signals are employed to elucidate the diads.

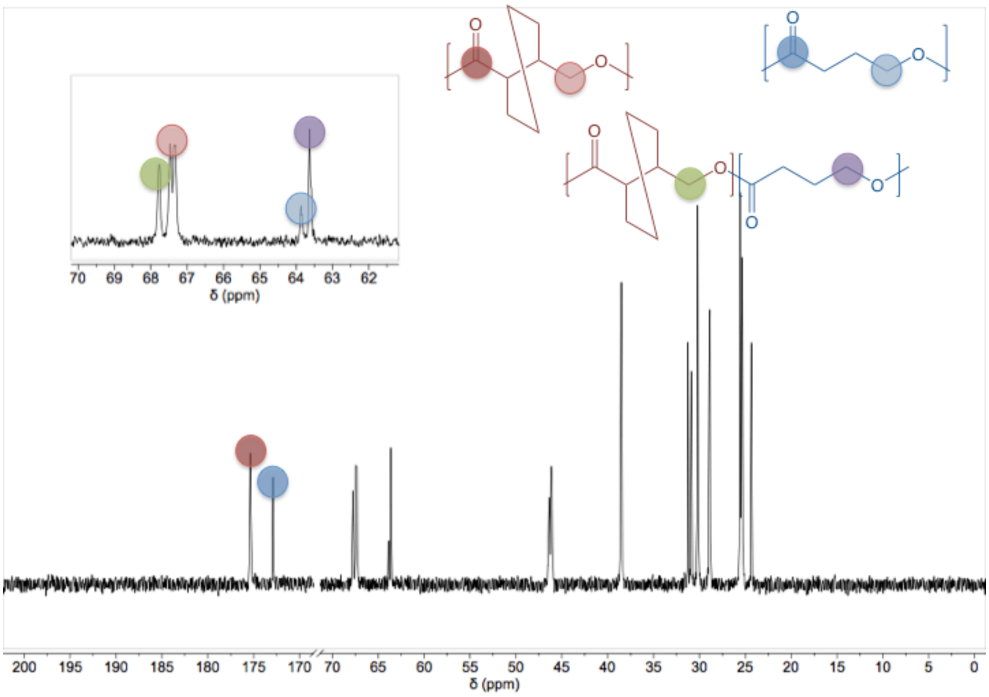

Supplementary Figure 12. ^{13}C NMR spectrum of PT6HP-*co*-PγBL 27 % γBL copolymer. Experimental conditions were as follows: 40 mg of sample, 25 °C, 1 s acquisition time, 2 s delay time, 10 µs pulse, spectral width 25900 Hz and more than 1100 scans. The samples were dissolved in deuterated chloroform. The colors in the signals correspond to the colors in the chemical structure and these signals are employed to elucidate the diads.

Supplementary Table 7. Microstructure of PT6HP-*co*-PγBL copolymers: number-average sequence lengths of T6HP and γBL, l_i, and the randomness character, η

Samples	l_{T6HP}	l_{GBL}	η
PT6HP-*co*-PγBL 7 %	14.8	1.3	0.82
PT6HP-*co*-PγBL 18 %	5.4	1.2	1.03
PT6HP-*co*-PγBL 27 %	3.6	1.4	1.02

Supplementary Table 8. Thermal properties of the synthetized polymers and copolymers

Samples	T_g (°C)	T_m (°C)	ΔH_m (Jg^{-1})	$T_{d,5\%}$ (°C)	T_{max} (°C)
PT6HP	49	-	-	342	391
PT6HP-*co*-PγBL 7 %	49	-	-	346	374
PT6HP-*co*-PγBL 18 %	34	-	-	297	370
PT6HP-*co*-PγBL 27 %	23	-	-	257	296, 351
PγBL	-45	58.2	41	218	201

T_g from the first DSC heating scan.

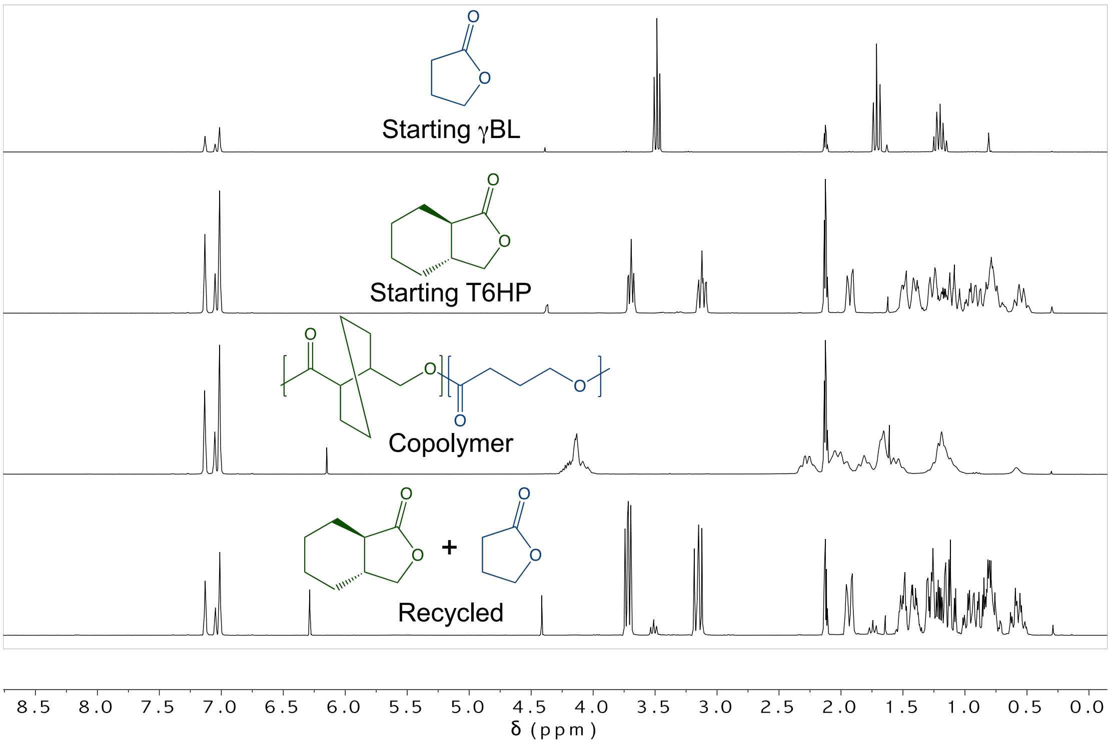

Supplementary Figure 13. Depolymerization of a copolymer film. The film (0.235 g) was depolymerized following a similar procedure in a Schlenk tube employing non deuterated toluene (4 mL). For the NMR analysis an aliquot was extracted and deuterated toluene was added.

Supplementary References

[1] Zhu, J.-B., Watson, E.M., Tang, J. & Chen, E.Y.-X. A synthetic polymer system with repeatable chemical recyclability. *Science* **360**, 398-403 (2018).

[2] Cheng, M., Attygalle, A.B., Lobkovsky, E.B. & Coates, G.W. Single-site catalysts for ring-opening polymerization: synthesis of heterotactic poly(lactic acid) from rac-Lactide. *J. Am. Chem. Soc.* **121**, 11583-11584 (1999).

[3] Amgoune, A., Thomas, C.M., Roisnel, T. & Carpentier, J.F. Ring-opening polymerization of lactide with group 3 metal complexes supported by dianionic alkoxy-amino-bisphenolate ligands: combining high activity, productivity, and selectivity. *Chem. Eur. J.* **12**, 169-179 (2005).

[4] Miguel, O., Iruin, J.J. & Fernandez-Berridi, M.J. Survey on transport properties of liquids, vapors, and gases in biodegradable poly(3-hydroxybutyrate) (PHB). *J. Appl. Polym. Sci.* **64**, 1849–1859 (1997).

[5] Yampolskii, Y., Pinnau, I. & Freeman, B.D. *Materials Science of Membranes* (John Wiley & Sons Ltd., London, 2006).

[6] Ibbet, R.N. *NMR Spectroscopy of Polymers* Ch. 2 (Blackie Academic & Professional, London, 1993).

[7] Chaos, A. et al. Tributyl citrate as effective plasticizer for biodegradable polymers: effect of plasticizer on free volume and transport and mechanical properties. *Polym. Int.* **68**, 125-133 (2019).

[8] Karayannidis, G.P., Sideridou, I.D., Zamboulis, D.N., Bikiaris, D.N. & Sakalis, A.J. Thermal behavior and tensile properties of poly(ethylene terephthalate-co-ethylene isophthalate). *J. Appl. Polym. Sci.* **78**, 200-207 (2000).

[9] Jokar, M., Rahman, A.R., Ibrahim, N.A., Abdullah, L.C. & Tan, C.P. Melt production and antimicrobial efficiency of low-density polyethylene (LDPE)-silver nanocomposite film. *Food Bioproc. Tech.* **5**, 719-728 (2012).

[10] Sangroniz, A. et al. Influence of the rigid amorphous fraction and crystallinity on polylactide transport properties. *Macromolecules*, **51**, 3923-3931, (2018).

[11] Sangroniz, L. et al. Polyethylene terephthalate/low density polyethylene/titanium dioxide blend nanocomposites: morphology, crystallinity, rheology, and transport properties. *J. Appl. Polym. Sci.* **136**, 46986 (2019).

[12] Brolly, J.B., Bower, D.I. & Ward, I.M. Diffusion and sorption of CO_2 in poly(ethylene terephthalate) and poly(ethylene naphthalate). *J. Polym. Sci., Part B: Polym. Phys.* **34**, 769-780 (1996).
